# The Application of CA and PCA to the Evaluation of Lipophilicity and Physicochemical Properties of Tetracyclic Diazaphenothiazine Derivatives

**DOI:** 10.1155/2019/8131235

**Published:** 2019-10-20

**Authors:** Anna Nycz–Empel, Katarzyna Bober, Mirosław Wyszomirski, Ewa Kisiel, Andrzej Zięba

**Affiliations:** ^1^Department of Organic Chemistry, Faculty of Pharmaceutical Sciences in Sosnowiec, Medical University of Silesia in Katowice, Jagiellonska 4, 41-200 Sosnowiec, Poland; ^2^Deparment of Analytical Chemistry, Faculty of Pharmaceutical Sciences in Sosnowiec, Medical University of Silesia in Katowice, Jagiellonska 4, 41-200 Sosnowiec, Poland; ^3^University of Bielsko-Biala, Faculty of Materials, Civil and Environmental Engineering, Willowa 2, 43-309 Bielsko-Biala, Poland

## Abstract

The subject of the study was 11 new synthetized tetracyclic diazaphenothiazine derivatives. Using thin-layer chromatography in a reverse phase system (RP-TLC), their *R*_M0_ lipophilicity parameter was determined. The mobile phase was composed of 0.2 M Tris buffer (pH = 7.4) and acetone (POCH S.A., Gliwice, Poland) in different concentrations. Using computer programs, based on different computational algorithms, theoretical values of lipophilicity (AClogP, ALOGP, ALOGPs, miLogP, MLOGP, XLOGP2, and XLOGP3) as well as molecular descriptors (molecular weight, volume of a molecule, dipole moment, polar surface, and energy of HOMO orbitals and LUMO orbitals) and parameters of biological activity: human intestinal absorption (HIA), plasma protein binding (PPB), and blood-brain barrier (BBB), were determined. The correlations between the experimental values of lipophilicity and theoretically calculated lipophilic values and also between experimental values of lipophilicity and values of physicochemical or biological properties were assessed. A certain relationship between structure and lipophilicity was found. On the other hand, the relationships between *R*_M0_ and physicochemical or biological properties were not statistically significant and therefore unusable. For all analysed values, an analysis of similarities and principal component analyses were also made. The obtained dendrograms for the analysis of lipophilicity and physicochemical and biological properties indicate the relationship between experimental values of lipophilicity and structure in the case of theoretical lipophilicity values only. PCA, on the other hand, showed that ALOGP, MLOGP, miLogP, and BBB and molar volume have the largest share in the description of the entire system. Distribution of compounds on the area of factors also indicates the connections between them related to their structure.

## 1. Introduction

Designing new compounds which could find the application as drugs (or their precursors) is an expensive and time-consuming process. There is ongoing research concerning a new solution that could limit the number of necessary syntheses and at the same time obtain the products with optimal physicochemical properties correlated with biological activity [1]. QSAR (quantitative structure-activity relationship) is the leading method used in obtaining new drugs. It describes the quantitative relationship between the necessary biological activity of the compound and its chemical structure [2, 3]. To define the differences between a series of similar substances, the QSAR descriptors are used. They are physicochemical parameters that are an interpretation of the biological properties of the compounds investigated [4]. One of the most often used parameters in QSAR analysis is lipophilicity. It can be connected with all drug interaction phases in the body, i.e., pharmaceutical, pharmacokinetic, and pharmacodynamic phases [5]. In the pharmaceutical phase, it affects the form of the drug, the way it is administered, and the release in the body. Also, the processes of absorption and distribution of biologically active substances depend significantly on lipophilicity, which is the main factor determining the bioavailability of the drug, and therefore its solubility in bodily fluids and the ease with which it is transported through biological membranes. Moreover, the lipophilic properties of the drug substance affect the way it interacts with the target receptor, and thus the pharmacological effect [6]. Lipophilicity determines the affinity of the molecule to the organic phase. It is expressed as the partition of substances in a two-phase system: liquid-liquid or liquid-solid; most often, lipophilicity is described by partition processes between polar (organic) and nonpolar (water) phases [7]. The traditional method of its determination is extraction in the octanol-water system [8, 9]. Currently, the chromatographic methods in which the test substance undergoes partition in a dynamic system composed of the mobile and stationary phases are of great importance in the determination of lipophilicity parameters [10, 11]. This method precisely and simultaneously determines many test substances, whose distribution coefficient can be in a wide range. In thin-layer chromatography and high-performance liquid chromatography, octadecylsilanized silica gel (RP-18) is used as the stationary phase, which to some extent imitates the structure of long-chain fatty acids in biological membranes. The mobile phase is usually the water-organic solution [11–13]. One of the groups of the drugs that are important from the point of view of medicinal chemistry is phenothiazine neuroleptics, known since the 1950s. Initially, these compounds were used in psychiatry as antipsychotics [14, 15]. The basic structural unit of neuroleptic phenothiazine is a tricyclic system in which two benzene rings are connected by a nitrogen and sulfur atom to form a 1,4-thiazine ring, with an attached alkyl chain in the N-10 position [16]. It is a highly lipophilic system, which is related to the strong affinity of the phenothiazine molecule to the lipid bilayer of cell membranes of neurons and other lipid-rich tissues. This property of phenothiazines allows them to penetrate the blood-brain barrier, which determines the mechanism of neuroleptic action of these compounds [17, 18]. Modification of the basic tricyclic phenothiazine structure can be accomplished by introducing new pharmacophore substituents into the thiazine ring or benzene rings and by replacing the benzene rings with heterocyclic systems, e.g., pyridine, quinoline, or pyrimidine. These types of structure transformations can lead to new compounds with a different direction and impact force [19, 20]. Literature reports indicate a number of promising types of biological activity of classical phenothiazines and their new derivatives, including antibacterial [21–24], antifungal [25], antiprional [26], antiprotozoal [27, 28], and antiviral [29, 30]. In addition, the antitumor properties of phenothiazines and their ability to modify multidrug resistance of certain tumor cell lines have been described [31, 32]. A new group of phenothiazine derivatives is tetracyclic quinobenzothiazine derivatives, which were obtained by replacing one of the benzene rings with a quinoline ring [33, 34]. For a number of derivatives of this type, the lipophilicity parameters *R*_M0_ and log *P*_TLC_ (theoretical and experimental) were determined [35]. Moreover, their interesting anticancer properties have been described in relation to human tumor cells of the MDA-MB-231 line (breast cancer), SNB-19 (brain glioma), and C-32 (cutaneous melanoma) [36]. Using the conversion of the benzene ring in the quinobenzothiazine system to the pyridine ring, a number of new compounds have been synthetized containing different substituents at various positions of the pyridine ring. The aim of this work is to determine the lipophilicity parameters (*R*_M0_, log *P*_TLC_, and log *P*_calc_) of 15 new pyridoquinothiazine salts by thin-layer chromatography in the reverse phase of RP-TLC and using computational methods and correlating them with each other. In addition, the *R*_M0_ lipophilicity parameter will be correlated with other molecular descriptors, and with theoretical values of biological activity.

## 2. Materials and Methods

### 2.1. Chemicals

**Figure 1:**
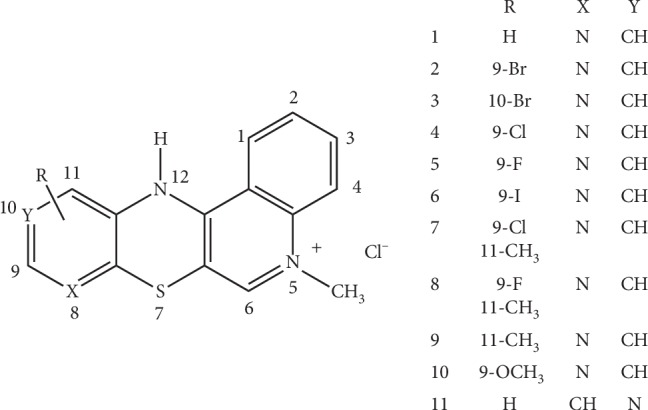
General structure of pyridoquinothiazine derivatives investigated (**1**–**11**).

The series of pyridoquinothiazine derivatives, described by symbols **1**–**11**, were investigated. The structures of compounds are shown in Figure 1.

Derivatives **1**–**11** were obtained through synthesis of thiochininantrenodiic chloride with a series of substituted aminopyridine derivatives. The methodology was described in our previous work [37]. The basic structure of the unsubstituted pyridoquinothiazine salt containing the nitrogen atom at position 8 of the tetracyclic system was modified by introducing various types of electron-withdrawing substituents and electron donors (Br, Cl, F, I, CH_3_, and OCH_3_) in positions 9, 10, and 11 of the pyridine ring. The previously unsubstituted **11** derivative has a nitrogen atom at position 10 of the pyridoquinothiazine system. The compounds have an additional methyl group on the pyridine or imine nitrogen atom. The structure of all compounds was confirmed by ESI-HRMS spectrometry and ^1^H, ^13^C NMR spectroscopy, using two-dimensional techniques HSQC, HMBC, NOESY, and COSY.

### 2.2. Thin-Layer Chromatography

The lipophilicity parameters were experimentally determined by reverse phase thin-layer chromatography (RP-TLC). Chromatograms were prepared on RP-18F_254s_ plates (1.05559.0001, Merck, Germany) precoated with nonpolar silicone oil. Plates were developed in glass chromatography chambers (Chromdes, Lublin, Poland) previously saturated with the vapour of the mobile phase. Solutions of pyridoquinothiazine salts **1**–**11** were prepared by dissolving 1 mg of particular compound in 2 ml of ethanol (POCH S.A., Gliwice, Poland). The solution was spotted into chromatographic plates in the amount of 2 *μ*L by use of microcapillary. The mobile phase was composed of 0.2 M Tris buffer (pH = 7.4) and acetone (POCH S.A., Gliwice, Poland) in different concentrations, i.e., 50%, 60%, 70%, 80%, and 90%. The chromatograms were visualised in UV light (*λ* = 254 nm). Determination of the *R*_F_ coefficient was carried twice for all compounds and in all acetone concentrations used. The final value of *R*_F_ is the mean of the two measurements. The obtained *R*_F_ coefficient was used to calculate the value of the *R*_M_ parameter, according to the following equation:

(1) $$\begin{array}{c} R_{\text{M}}=\log\left[\left(\frac{1}{R_{\text{f}}}\right)-1\right]. \end{array}$$

By extrapolating *R*_M_ values to zero acetone concentration, a relative *R*_M0_ lipophilicity parameter was obtained, according to the following equation:

(2) $$\begin{array}{c} R_{\text{M}}=R_{\text{M0}}+bC, \end{array}$$

where *b* is the slope and *C* is the concentration of acetone in mobile phase.

### 2.3. Computational Programs

For pyridoquinothiazine derivatives **1**–**11**, the theoretical values of the log *P*_calc_ parameter were determined using computer modules whose calculations are based on atoms, molecular fragments, and molecular properties (AClogP, ALOGP, ALOGPs, miLogP, MLOGP, XLOGP2, and XLOGP3) [38, 39]. The calculation methods used for obtaining the log *P* values were described earlier in our work concerning another group of new synthetized compounds [40]. Molecular descriptors (molecular weight, volume of a molecule, dipole moment, polar surface, and energy of HOMO orbitals and LUMO orbitals) were calculated using the DFT (density functional theory) method. A hybrid B3LYP function was used. Parameters of biological activity: human intestinal absorption (HIA), plasma protein binding (PPB), and blood-brain barrier (BBB), were determined using virtual computational bases [41].

### 2.4. Correlation, Cluster Analysis, and Principal Component Analysis

Based on the values of experimental lipophilicity (*R*_M0_, log *P*_TLC_) and theoretical parameters (log *P*_calc_), as well as the determined values of molecular descriptors and parameters of the predicted biological activity, correlation analysis, cluster analysis (CA), and principal component analysis (PCA) were performed. All data used for CA and PCA were standardised. The cluster analysis was based on the Euclidean distance, a single linkage method. The PCA analysis was based on the correlation matrix, using the Kaiser criterion and scree plot. The entire analysis was carried out using the Statistica 13.1 software.

## 3. Results and Discussion

The values of log *P*_TLC_, for compounds investigated, were calculated on the basis of known values of *R*_M0_, which were obtained by chromatographic analysis. In order to obtain the values of the log *P*_TLC_ parameter for the tested derivatives **1**–**11**, first, the analysis of standard substances with the well-known lipophilicity value (log *P*_lit_) was performed. The analysis was carried out under the same chromatographic conditions as for the tested substances. The reference compounds analysed were acetanilide **I**, p-cresol **II**, p-bromoacetophenone **III**, benzophenone **IV**, and anthracene **V**. Values of lipophilicity parameters of reference substances: literature (log *P*_lit_), experimental (*R*_M0_), and log *P*_TLC_, are presented in Table 1. Differences between the value of log *P*_lit_, and log *P*_TLC_ for the standards did not exceed ±0.190 and were below ±0.150 for three compounds.

Using the log *P*_lit_ relation from the experimentally determined *R*_M0_ parameters for the standards, a calibration curve was made. The linear function describing the calibration curve led to the formulation of the equation according to which log *P*_TLC_ was determined for the tested derivatives:

(3) $$\begin{array}{c} \log\;P_{\text{TLC}}=1.4664R_{\text{M0}}+0.1043\;\left(r=0.993,S\;D=0.162,F=230.9\right). \end{array}$$

The *R*_M0_ parameter values and log *P*_TLC_ values for derivatives **1**–**11** are shown in Table 2.

The log *P*_TLC_ parameters for the tested derivatives were within the range of 2.92–5.78. The collected results indicate the dependence of lipophilicity on the structure of the molecule as well as the presence and type of substituents at various positions of the pyridoquinothiazine system. Compounds with only an additional alkyl group: 11-CH3 (**9**, log *P*_TLC_: 4.19) and 9-OCH_3_ (**10**, log *P*_TLC_: 4.22), were characterised by lower values of lipophilicity. The introduction of the halogen atom in the system containing the −11 methyl group resulted in a significant increase in the log *P*_TLC_ parameter in relation to the initial compound, as observed for the derivatives **7** (9-Cl, log *P*_TLC_: 5.72) and **8** (9-F, log *P*_TCL_: 5.78). A significant reduction in lipophilicity was noted for the unsubstituted derivative **1** (log *P*_TLC_: 3.53) and for the isomeric derivative **11** (log *P*_TLC_: 2.92) in which the nitrogen atom of the pyridine ring is located in position 10 of the diazaphenothiazine system.

One of the stages of the research was the determination of the log *P* parameter using computer methods. Depending on the mathematical module of the program used, the obtained values of the log *P*_calc_ parameter occurred in a very wide range from −1.16 to 4.70 (Table 3).

It was not possible to obtain a difference fewer than 0.5 units between the parameters calculated log *P*_calc_ and experimental log *P*_TLC_ for all tested compounds in any of the programs used. Differing values of results stem from the method of counting lipophilicity. The computer programs calculating the lipophilicity parameters are based on the structure of neutral molecules. It does not take into account the influence of conformation, tautomerisation, ionisation, changes in electron density, or the formation of hydrogen bondsor ion pairs through compounds investigated. Moreover, literature reports indicate a significant influence of changes in the thiazine ring conformation and changes in electron density at individual nitrogen atoms on the lipophilicity of quinobenzothiazine tetradenic derivatives [45].

In order to determine the relationship between experimental and theoretically calculated parameters, a correlation analysis was performed for the *R*_M0_ and log *P*_calc_ parameters. In the studied group of derivatives, high and very high values of correlation coefficients were obtained for all computer modules used (*r* = 0.7555–0.9604, *p* < 0.05). Also, the log *P* values analysed correlated well with each other. With respect to these results, it can be stated that there is a relationship between the structure and the lipophilicity of the compounds tested. The correlation equations obtained are presented in Table 4.

As part of the work, attempts were made to correlate the lipophilicity parameters of derivatives **1**–**11** with the values of physicochemical properties such as the molar mass, volume of the molecule, dipole moment, polar surface, and HOMO-LUMO gap (Table 5).

In the group of analysed compounds, the best correlation of the *R*_M0_ parameter was obtained for the relation with the volume of the molecule (*r* = 0.8225). Statistically significant correlations were also obtained for relations with dipole moment (*r* = 0.6626) and with gap energy (*r* = 0.6496). Correlations with other physicochemical properties were statistically insignificant.

As mentioned earlier, lipophilicity has a significant impact on the behaviour of biologically active compounds in the human body, including their absorption when taken orally, binding to proteins in the bloodstream and penetration of the blood-brain barrier or blood-placenta barrier. Therefore, it seemed appropriate to carry out a correlation study of the relative lipophilicity parameter *R*_M0_ with the calculated HIA parameter (human intestinal absorption), the PPB parameter (plasma protein binding), and the BBB parameter (blood-brain barrier–blood-brain penetration factor). The values of biological activity coefficients are presented in Table 6.

The results suggest very good oral bioavailability of all test compounds **1**–**11** (HIA > 96%) (Table 6). The blood-brain barrier penetration ratio in both groups is also high for newly obtained salts and is in the range of 89.51–97.28%, which classifies them as potential neuroleptic drugs. The highest BBB parameter value was calculated for derivative **10** containing a 9-methoxy group (BBB = 97.28%). The degree of protein binding is more diverse and depends on the structure of the compound being analysed. For salts **1**–**11,** it ranged from 20.32 to 88.16%. The theoretical value of this coefficient affects the concentration of the free fraction of the compound in the bloodstream and thus its biological activity: the higher the PPB, the lower the biological activity. The lowest values of this parameter were noted for the isomeric derivative **11** (PPB = 20.32%). The correlation of the *R*_M0_ parameter with the calculated parameters of biological activity resulted in different regression coefficients. In the group of analyzed compounds, only for the relationship with the PPB parameter, a high positive statistically significant correlation was obtained (*r* = 0.7284). The remaining results indicate that there is no significant relationship between the analysed features; hence, correlation equations describing such relationships cannot be used to determine the *R*_M0_ parameter. Taking into account the above results, Table 7 presents correlation equations describing the relationships between lipophilicity values and physicochemical or biological parameters, but only those which are statistically significant.

**Figure 2:**
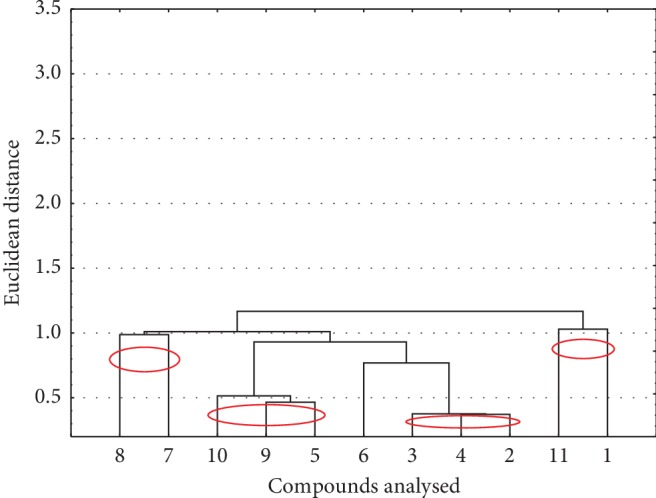
Similarity analysis for compounds investigated based on their values of lipophilicity.

For an additional description of the analysed compounds, the similarities were analysed, taking into account the lipophilicity values separately (experimental and theoretically calculated), values of physicochemical properties, and HIA, PPB, and BBB values. The obtained results are presented in the form of dendrograms. The first dendrogram (Figure 2) presents the similarity between analysed compounds based on their values of lipophilicity.

As a result of the analysis of similarities of lipophilicity values for the analysed compounds, several clusters are observed. Compounds 7 and 8 contain a hydrophobic methyl group at position 11. Moreover, they contain also the highly ellectronically accepting atoms at position 9, F in the case of compound 8 and Cl in the case of compound 7. Compounds **5**, **9,** and **10** contain electronegative F atom at position 9 and a methyl or methoxy group. The compounds **2**, **3,** and **4** contain in their structure, in the 9 or 10 position, a Cl or Br atom. The last cluster is formed by compounds **1** and **11**. They do not contain any additional substituents in the pyridine ring. In this case, it can be assumed that the change in the position of the N atom from position 8 to 9 has no significant effect on lipophilicity.

**Figure 3:**
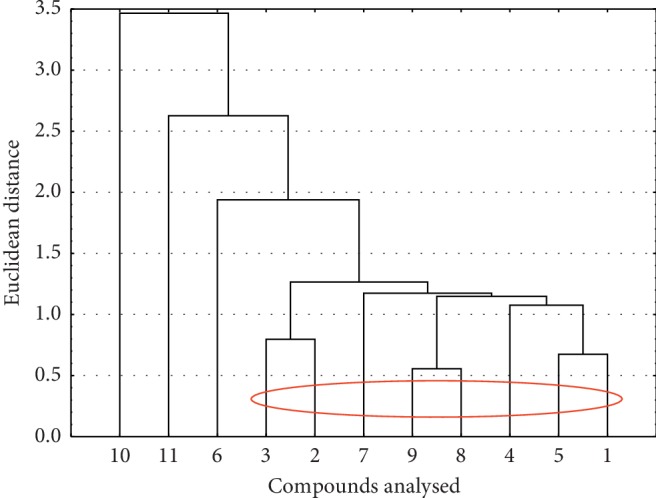
Similarity analysis for compounds investigated based on their values of physicochemical properties.

The second similarity analysis was done for values of physicochemical properties for all compounds investigated (Figure 3).

Based on the data analysis of the physicochemical properties, there is one large cluster containing all of the compounds tested, except for 6, 10, and 11. This may be due to the fact that compound **6** has an iodine atom with a large volume in its structure, whereas compound **10**—methoxy group—and compound **11** have a nitrogen atom at position 10 of the pyridine ring. The remaining compounds show great similarity taking into consideration the physicochemical properties.

**Figure 4:**
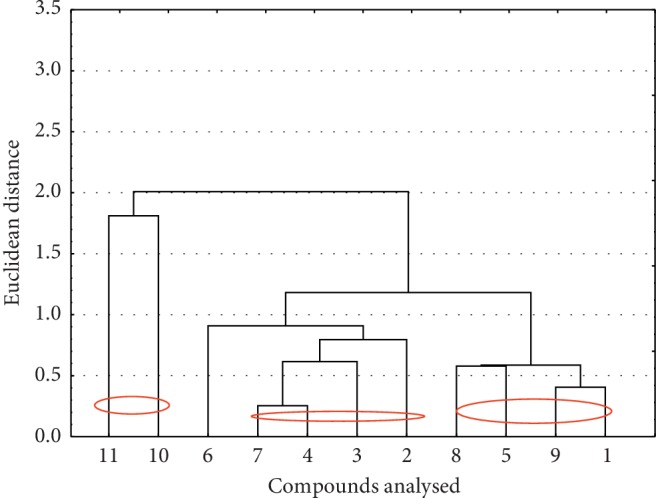
Similarity analysis for compounds investigated based on their values of biological properties.

The next similarity analysis was done for values of biological properties (HIA, PPB, and BBB) for all compounds investigated (Figure 4).

Several clusters were obtained as a result of the analysis. Compounds **2**, **3**, **4,** and **7** contain a Cl or Br atom in positions 9 and 10. In addition, it turned out that compounds **10** and **11** are similar in biological properties, while compounds **1**, **5**, **8,** and **9** are the most similar and form a separate cluster. It should also be emphasised that the compounds investigated show the largest similarity when only the lipophilicity (theoretical and experimental) values were taken into account. For them, the Euclidean distance was the smallest.

**Figure 5:**
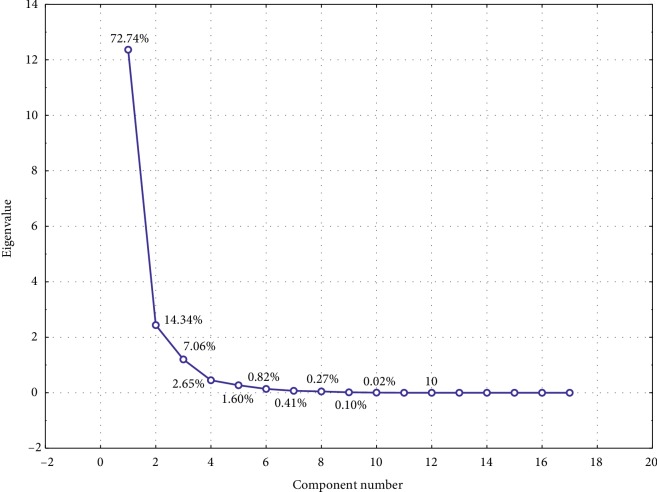
Scree plot for experimental and theoretical data analysed.

For a better description of the obtained experimental and theoretical data for the tested compounds, the principal components analysis (PCA) was also used. Eigenvalues were extracted based on all data. Three main components were selected using the Kaiser criterion, the value of which exceeds 1. These selected components in 94.13% describe the variability of the system. However, when analysing the scree plot presented in Figure 5, it can be concluded that the main components should be 4. Then, the variability of the system would be described in almost 97%.

**Figure 6:**
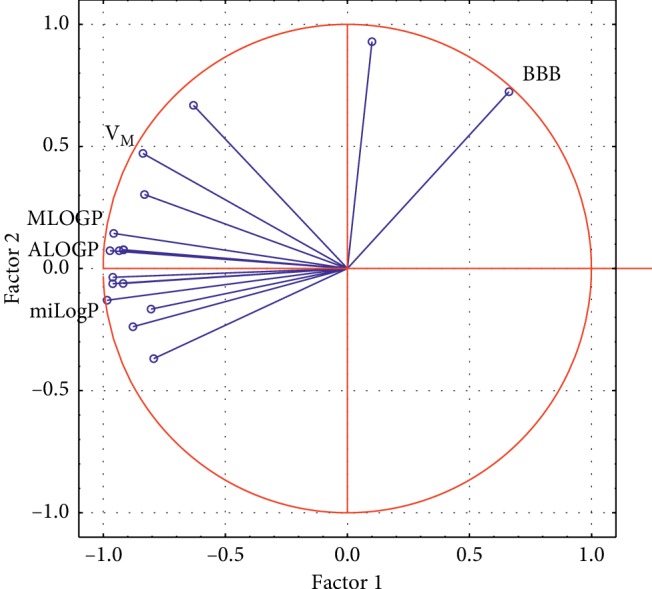
Projection of variables on the area of factors based on the first two eigenvalues.

Figure 6 presents a view of variables on the area of factors, showing the share of individual variables in the main components. The longest vectors showing the largest share are characterised by the following variables: ALOGP, MLOGP, miLogP, and BBB and molar volume. Most of these variables are closely related to the structure of the analysed compounds, which was confirmed by previous analyses.

**Figure 7:**
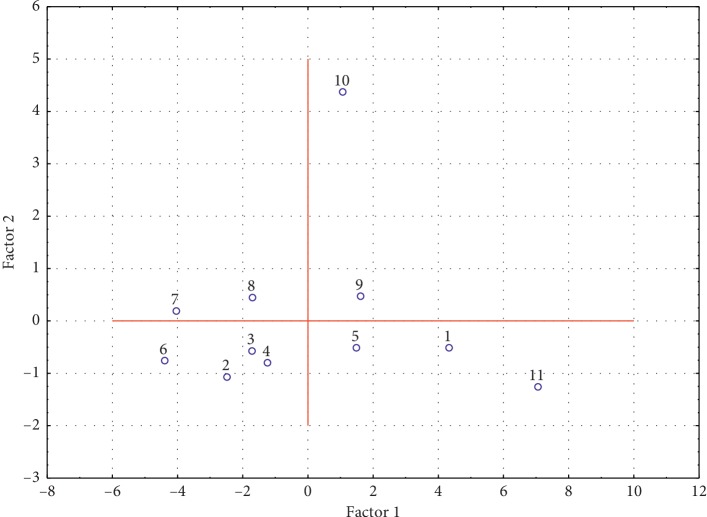
The distribution of cases (compounds) on the area of factors.

Through PCA analysis, the distribution of cases (compounds) on the area of factors can be presented (Figure 7).

It is clearly visible that the analysed compounds practically form one group, with the exception of compound **10**. It can most certainly be related to the structure of the compound, as compound **10** is the only one with a methoxy group in position 9 in its structure. The location of compounds **1** and **11** in one place may also indicate a connection with their structure because only they, from all tested compounds, have no substituents.

## 4. Conclusion

The subject of the study was new synthetized tetracyclic diazaphenothiazine derivatives. Using thin-layer chromatography in an inverse phase system (RP-TLC), the *R*_M0_ lipophilicity parameter for these was determined. Using computer programs, based on different computational algorithms, theoretical, mainly based on compound structure, lipophilicity values, as well as physicochemical and biological properties were determined. It can be concluded, through analysis of the obtained correlations between the experimental values of lipophilicity and the theoretically calculated lipophilic values, that there is a certain relationship between structure and lipophilicity. The relationships between *R*_M0_ and ALOGP and AClogP values are characterised by high values of correlation coefficients, 0.9604 and 0.9382, respectively. On the other hand, relationships between *R*_M0_ and physicochemical or biological properties were not statistically significant and therefore unusable. For all analysed values, an analysis of similarities and principal component analyses were also made. The obtained dendrograms for the analysis of lipophilicity and physicochemical and biological properties indicate the relationship between experimental values of lipophilicity and structure, but only in the case of theoretical lipophilicity values. PCA, on the other hand, showed that ALOGP, MLOGP, miLogP, and BBB and molar volume have the largest share in the description of the entire system. The distribution of compounds on the area of factors also indicates the connections between them related to their structure.

## Figures and Tables

**Table 1:** Values of log *P*_lit_ parameters, experimentally determined *R*_M0_, and log *P*_TLC_ for reference substances **I**–**V**.

Lipophilicity	Standard substances				
	I [42]	II [43]	III [44]	IV [42]	V [44]
log *P*_lit_	1.2100	1.9700	2.4300	3.1800	4.4500
*R *_M0_	0.6800	1.2930	1.6260	2.2240	2.8500
−*b*	0.0119	0.0196	0.0207	0.0280	0.0335
*r*	0.9908	0.9953	0.9945	0.9985	0.9989
SD	0.0296	0.0346	0.0398	0.0278	0.0282
log *P*_TLC_	1.0670	2.0380	2.5090	3.3630	4.2610

*b*: slope; *r*: correlation coefficient; SD: standard error.

**Table 2:** The values of the *R*_M0_ parameter obtained on the basis of the equation *R*_M_ = *R*_M0_ + (*b)C* and the value of log *P*_TLC_ for the **1**–**11** derivatives.

No	*R*_M0_	−*b*	*r*	SD	log *P*_TLC_
**1**	2.3379	0.0278	0.9957	0.0470	3.53
**2**	3.2293	0.0391	0.9924	0.0882	4.83
**3**	3.0699	0.0361	0.9900	0.0935	4.61
**4**	3.1016	0.0377	0.9907	0.0943	4.65
**5**	2.8262	0.0343	0.9946	0.0648	4.25
**6**	3.4373	0.0410	0.9900	0.1062	5.14
**7**	3.8299	0.0450	0.9934	0.0943	5.72
**8**	3.8738	0.0463	0.9913	0.1122	5.78
**9**	2.7871	0.0333	0.9906	0.0850	4.19
**10**	2.8087	0.0325	0.9941	0.0649	4.22
**11**	1.9173	0.0241	0.9961	0.0386	2.92

**Table 3:** Values of lipophilicity parameters of 5-methyl-12 (*H*)-quin [3,4-*b*] pyrido [2,3-*e*] [1, 4] thiazine salts 1–11 obtained using computer methods.

No.	AClogP	ALOGP	ALOGPs	miLogP	MLOGP	XLOGP2	XLOGP3
1	2.74	3.35	−0.90	−0.13	1.38	2.69	3.27
2	3.35	4.33	−0.12	1.02	2.02	3.58	4.30
3	3.44	4.09	−0.30	0.83	2.02	3.49	3.96
4	3.44	4.22	−0.15	0.89	1.90	3.40	4.23
5	3.28	3.93	−0.42	0.38	1.78	2.94	3.71
6	3.76	4.45	−0.13	1.30	2.14	3.85	3.96
7	3.76	4.70	0.01	1.27	2.14	3.63	4.60
8	3.59	4.42	−0.28	0.75	2.02	3.17	4.07
9	3.06	3.83	−0.75	0.25	1.63	2.92	3.64
10	3.11	3.87	−0.58	0.27	1.91	3.34	3.58
11	2.65	2.81	−1.16	−0.33	1.38	2.61	2.94

**Table 4:** Correlation equations for relationships between *R*_M0_ and lipophilicity values theoretically calculated for compounds **1**–**11**, *p* < 0.05.

	Correlation equation	*r*
AClogP	*R*_M0_ = 1.4803 log *P*_calc_ − 1.8488	0.9382
ALOGP	*R*_M0_ = 1.0402 log *P*_calc_ − 1.1408	0.9604
ALOGPs	*R*_M0_ = 1.4052 log *P*_calc_ + 3.6305	0.8901
miLogP	*R*_M0_ = 0.9497 log *P*_calc_ + 2.4587	0.8811
MLOGP	*R*_M0_ = 1.9049 log *P*_calc_ − 0.499	0.8944
XLOGP2	*R*_M0_ = 1.0943 log *P*_calc_ − 0.5237	0.7555
XLOGP3	*R*_M0_ = 1.0971 log *P*_calc_ − 1.195	0.8950

**Table 5:** Values of physicochemical properties for derivatives **1**–**11**.

No.	*M*_M_	*μ* (D)	*V*_M_ (Å^3^)	PSA (Å^2^)	HOMO (eV)	LUMO (eV)	Gap (eV)
**1**	266.342	12.1387	236.8	32.57	−0.323134	−0.220941	−0.10219
**2**	345.236	12.355	254.69	32.57	−0.324275	−0.224889	−0.09939
**3**	345.236	13.4737	254.69	32.57	−0.325208	−0.225792	−0.09942
**4**	300.785	12.4105	250.34	32.57	−0.324502	−0.223992	−0.10051
**5**	284.330	12.4232	241.73	32.57	−0.327587	−0.225137	−0.10245
**6**	392.236	13.2687	260.79	32.57	−0.320785	−0.225700	−0.09509
**7**	314.811	13.7753	266.9	32.57	−0.32108	−0.222058	−0.09902
**8**	298.357	13.8931	258.29	32.57	−0.324334	−0.222905	−0.10143
**9**	280.367	13.6158	256.36	32.57	−0.319921	−0.21898	−0.10094
**10**	296.366	14.8075	262.35	41.80	−0.312113	−0.213951	−0.09816
**11**	266.339	9.4344	230.61	32.57	−0.331136	−0.223702	−0.10743

**Table 6:** Values of factors of biological activity (HIA, PPB, and BBB) for compounds **1**–**11**.

No.	HIA	PPB	BBB
1	96.36	58.00	92.86
2	96.96	88.25	90.18
3	96.96	69.39	90.18
4	96.75	78.07	90.93
5	96.36	54.44	91.51
6	97.23	89.16	89.51
7	96.82	76.16	90.76
8	96.46	64.04	91.44
9	96.45	63.65	92.76
10	96.35	45.74	97.28
11	96.35	20.32	94.38

**Table 7:** Correlation of the *R*_M0_ parameter with physicochemical properties (dipole moment *μ*, volume of molecule *V*_M_, and HOMO-LUMO energy difference (gap)) and PPB for derivatives **1**–**11**, *p* < 0.05.

Structural descriptor	Correlation equation	*r*
*μ*	*R*_M0_ = 0.2764*μ* − 0.5379	0.6626
*V*_M_	*R*_M0_ = 00426*V*_M_ − 7.7315	0.8225
Gap	*R*_M0_ = 123.3884gap + 15.4264	0.6495
PPB	*R*_M0_ = 0.0214PPB% + 1.6425	0.7284

## Data Availability

All chromatographic data used to support the findings of this study are available from the corresponding author upon request.

## References

[1] Hughes J. P., Rees S., Kalindjian S. B., Philpott K. L. (2011). Principles of early drug discovery. *British Journal of Pharmacology*.

[2] Schultz T. W., Cronin M. T. D., Walker J. D., Aptula A. O. (2003). Quantitative structure–activity relationships (QSARs) in toxicology: a historical perspective. *Journal of Molecular Structure: THEOCHEM*.

[3] Hansch C., Maloney P. P., Fujita T., Muir R. M. (1962). Correlation of biological activity of phenoxyacetic acids with Hammett substituent constants and partition coefficients. *Nature*.

[4] Xue L., Bajorath J. (2000). Molecular descriptors for effective classification of biologically active compounds based on principal component analysis identified by a genetic algorithm. *Journal of Chemical Information and Computer Sciences*.

[5] Arnott J. A., Planey S. L. (2012). The influence of lipophilicity in drug discovery and design. *Expert Opinion on Drug Discovery*.

[6] Jóźwiak K., Szumiło H., Soczewiński E. (2011). Lipofilowość, metody wyznaczania i rola w działaniu biologicznym substancji chemicznych. *Wiadomości Chemiczne*.

[7] Martin Y. C., Abagyan R., Ferenczy G. G. (2016). Glossary of terms used in computational drug design, part II (IUPAC Recommendations 2015). *Pure and Applied Chemistry*.

[8] Pliška V., Testa B., van de Waterbeemd H. (1996). Lipophilicity in drug action and toxicology. *Journal of Medicinal Chemistry*.

[9] Rutkowska E., Pajak K., Jóźwiak K. (2013). Lipophilicity—methods of determination and its role in medicinal chemistry. *Acta Poloniae Pharmaceutica*.

[10] Waring M. (2010). Liphophilicity in drug discovery. *Expert Opinion in Drug Discovery*.

[11] Giaginis C., Tsantili-Kakoulidou A. (2008). Alternative measures of lipophilicity: from octanol-water partitioning to IAM retention. *Journal of Pharmaceutical Sciences*.

[12] Hartmann T., Schmitt J. (2004). Lipophilicity—beyond octanol/water: a short comparison of modern technologies. *Drug Discovery Today: Technologies*.

[13] Paszkowska J., Kania B., Wandzik I. (2012). Evaluation of the lipophilicty of selected uridine derivatives by use of RP-TLC, shake-flask, and computational methods. *Journal of Liquid Chromatography & Related Technologies*.

[14] Gupta R. R., Kumar M., Gupta R. R. (1988). Synthesis, properties and reactions of phenothiazines. *Phenothazines and 1,4-Benzothiazines: Chemical and Biomedical Ascpects*.

[15] López-Muñoz F., Alamo C., Cuenca E., Shen W., Clervoy P., Rubio G. (2005). History of the discovery and clinical introduction of chlorpromazine. *Annals of Clinical Psychiatry*.

[16] Zejc A., Górczyca M. (2008). *Chemia Leków*.

[17] Mahajan S., Mahajan R. K. (2013). Interactions of phenothiazine drugs with surfactants: a detailed physicochemical overview. *Advances in Colloid and Interface Science*.

[18] Cheema M. A., Barbosa S., Taboada P., Castro E., Siddiq M., Mosquera V. (2006). A thermodynamic study of the amphiphilic phenothiazine drug thioridazine hydrochloride in water/ethanol solvent. *Chemical Physics*.

[19] Morak-Młodawska B., Jeleń M., Pluta K. (2009). Nowe pochodne fenotiazyn o właściwościach przeciwnowotworowych. *Polski Merkuriusz Lekarski*.

[20] Jaszczyszyn A., Gąsiorowski K., Świątek P. (2012). Chemical structure of phenothiazines and their biological activity. *Pharmacological Reports*.

[21] Zięba A., Czuba P., Król W. (2012). In vitro antimicrobial activity of novel azaphenothiazine derivatives. *Acta Poloniae Pharmaceutica–Drug Research*.

[22] Klitgaard J. K., Skov M. N., Kallipolitis B. H., Kolmos H. J. (2008). Reversal of methicillin resistance in *Staphylococcus aureus* by thioridazine. *Journal of Antimicrobial Chemotherapy*.

[23] Warman A. J., Rito T. S., Fisher N. E. (2013). Antitubercular pharmacodynamics of phenothiazines. *Journal of Antimicrobial Chemotherapy*.

[24] Dasgupta A., Mukherjee S., Chaki S. (2010). Thioridazine protects the mouse from a virulent infection by *Salmonella enterica* serovar typhimurium 74. *International Journal of Antimicrobial Agents*.

[25] Dastidar M. C., DiDone L., Heier R. F., Meyers M. J., Krysan D. J. (2018). Antifungal Phenothiazines: optimization, characterization of mechanism, and modulation of neuroreceptor activity. *ACS Infectious Diseases*.

[26] Korth C., May B. C. H., Cohen F. E., Prusiner S. B. (2001). Acridine and phenothiazine derivatives as pharmacotherapeutics for prion disease. *Proceedings of the National Academy of Sciences*.

[27] Barbosa A. F., Sangiorgi B. B., Galdino S. L., Barral-Netto M., Pitta I. R., Pinheiro A. L. (2012). Photodynamic antimicrobial chemotherapy (PACT) using phenothiazine derivatives as photosensitizers against *Leishmania braziliensis*. *Lasers in Surgery and Medicine*.

[28] Kim J.-H., Jung S.-Y., Lee Y.-J. (2008). Effect of therapeutic chemical agents in vitro and on experimental meningoencephalitis due to naegleria fowleri. *Antimicrobial Agents and Chemotherapy*.

[29] Perin P. M., Haid S., Brown R. J. P. (2016). Flunarizine prevents hepatitis C virus membrane fusion in a genotype-dependent manner by targeting the potential fusion peptide within E1. *Hepatology*.

[30] Chamoun-Emanuelli A. M., Pecheur E.-I., Simeon R. L., Huang D., Cremer P. S., Chen Z. (2013). Phenothiazines inhibit hepatitis C virus entry, likely by increasing the fluidity of cholesterol-rich membranes. *Antimicrobial Agents and Chemotherapy*.

[31] Motohashi N., Kawase M., Saito S., Sakagami H. (2000). Antitumor potential and possible targets of phenothiazine-related compounds. *Current Drug Targets*.

[32] Motohashi N., Kawase M., Satoh K., Sakagami H. (2006). Cytotoxic potential of phenothiazines. *Current Drug Targets*.

[33] Zięba A., Sochanik A., Szurko A., Rams M., Mrozek A., Cmoch P. (2010). Synthesis and in vitro antiproliferative activity of 5-alkyl-12(*H*)-quino[3,4-*b*][1,4]benzothiazinium salts. *European Journal of Medicinal Chemistry*.

[34] Zięba A., Latocha M., Sochanik A. (2013). Synthesis and in vitro antiproliferative activity of novel 12(*H*)-quino[3,4-*b*][1,4]benzothiazine derivatives. *Medicinal Chemistry Research*.

[35] Zięba A., Bober K. (2016). Liphophilicity of newly synthetized quinobenzothiazines by use of TLC. *Journal of Liquid Chromatography & Related Technologies*.

[36] Zięba A., Latocha M., Sochanik A., Nycz A., Kuśmierz D. (2016). Synthesis and in vitro antiproliferative activity of novel phenyl ring-substituted 5-alkyl-12(*H*)-quino[3,4-*b*][1,4]benzothiazine derivatives. *Molecules*.

[37] Zięba A., Suwińska K. (2006). 1-alkyl-4-(3-pirydynylamino)quinolinium-3-thiolates and their transformation into new diazaphenothiazine derivatives. *Heterocycles*.

[38] ClogP (C. S. Chem 3D Ultra 7.0. Molecular Modeling and Analysis) Distributed by Cambridge Soft

[39] VCCLAB (February 2019). Virtual computational chemistry laboratory. http://www.vcclab.org.

[40] Zięba A., Bober K. (2018). Application of thin-layer chromatography to the lipophilicity analysis of selected anticancer quinobenzothiazine derivatives. *Journal of Planar Chromatography*.

[41] http://preadmet.bmdrc.org/dt_benefits/adme-predicition

[42] Bodor N., Gabanyi Z., Wong C. K. (1989). A new method for the estimation of partition coefficient. *Journal of the American Chemical Society*.

[43] Dross K., Sonntag C., Mannhold R. (1994). Determination of the hydrophobicity parameter RMw by reversed-phase thin-layer chromatography. *Journal of Chromatography A*.

[44] Mannhold R., Cruciani G., Dross K., Rekker R. (1998). Multivariate analysis of experimental and computational descriptors of molecular liphophilicity. *Journal of Computer-Aided Molecular Design*.

[45] Zięba A., Prus W W. (2009). Determination of the liphopilicity of new azaphenothiazines by reversed-phase thin-layer chromatography. *Acta Chromatographica*.

